# Increased [^18^F]FDG uptake of radiation-induced giant cells: a single-cell study in lung cancer models

**DOI:** 10.1038/s44303-024-00017-3

**Published:** 2024-06-19

**Authors:** Neeladrisingha Das, Hieu T. M. Nguyen, Wan-Jin Lu, Arutselvan Natarajan, Syamantak Khan, Guillem Pratx

**Affiliations:** 1 Department of Radiation Oncology, Stanford University, Stanford, CA USA; 2 Institute for Stem Cell Biology and Regenerative Medicine, Stanford University, Stanford, CA USA; 3 Department of Radiology, Stanford University, Stanford, CA USA

**Keywords:** Cancer imaging, Cancer imaging

## Abstract

Positron emission tomography (PET), a cornerstone in cancer diagnosis and treatment monitoring, relies on the enhanced uptake of fluorodeoxyglucose ([^18^F]FDG) by cancer cells to highlight tumors and other malignancies. While instrumental in the clinical setting, the accuracy of [^18^F]FDG-PET is susceptible to metabolic changes introduced by radiation therapy. Specifically, radiation induces the formation of giant cells, whose metabolic characteristics and [^18^F]FDG uptake patterns are not fully understood. Through a novel single-cell gamma counting methodology, we characterized the [^18^F]FDG uptake of giant A549 and H1299 lung cancer cells that were induced by radiation, and found it to be considerably higher than that of their non-giant counterparts. This observation was further validated in tumor-bearing mice, which similarly demonstrated increased [^18^F]FDG uptake in radiation-induced giant cells. These findings underscore the metabolic implications of radiation-induced giant cells, as their enhanced [^18^F]FDG uptake could potentially obfuscate the interpretation of [^18^F]FDG-PET scans in patients who have recently undergone radiation therapy.

## Introduction

With its exceptional molecular sensitivity throughout the entire body, positron emission tomography (PET) has become an essential imaging tool for the diagnosis, staging, and monitoring of cancer. When combined with fluorodeoxyglucose ([^18^F]FDG), PET efficiently maps the heightened glucose metabolism commonly observed in cancer cells, allowing for effective detection, delineation, and quantification, of tumor regions^1,2^. However, while [^18^F]FDG-PET offers unparalleled insights into the metabolic activity of tumors, it also introduces unexpected complexities when used in conjunction with therapeutic interventions such as radiation therapy. While the primary aim of radiation is to damage the DNA of cancer cells and halt their proliferation, it can inadvertently induce an array of other cellular responses, including within metabolic pathways^3,4^. A prominent consequence of radiation therapy is the emergence of giant cells, which are characterized by multiple nuclei and are a direct result of radiation-induced disruptions in the cell cycle^5,6^. Though the proliferation potential of these giant cells is often lost, their metabolic activity, especially with respect to [^18^F]FDG uptake, can be significantly enhanced^7,8^.

This potential interaction between radiation-induced giant cells and [^18^F]FDG uptake is of significant clinical importance. The premise of [^18^F]FDG-PET relies on differential glucose metabolism to identify tumor regions^9,10^. If the metabolic signatures of these non-dividing, yet metabolically active giant cells are not thoroughly understood, a potential risk is that ambiguous or even misleading imaging results could be obtained through [^18^F]FDG-PET studies. To address this crucial gap in our understanding, we sought to delve deeper into the metabolic characteristics of radiation-induced giant cells in the context of [^18^F]FDG-PET imaging.

The A549 human lung carcinoma cell line was selected as a model of a radioresistant cancer for this study. Additionally, the H1299 lung cancer cell line was included to provide additional validation. As in the A549 model, many lung cancers harbor mutations in KEAP-1 that limit the efficacy of radiotherapy^11^. Early assessment of treatment response in these cancers could be achieved through interim PET imaging, provided the growth and metabolic characteristics of radiation-induced giant cells are understood. Towards this goal, we developed a novel single-cell assay to characterize these cells and their metabolic traits and measure [^18^F]FDG uptake on a single-cell level.

To extrapolate our in vitro findings to a more clinically relevant scenario, we repeated this analysis using cells derived from an in vivo tumor xenograft model based on the A549 cell line. Using this model, we comprehensively assessed how these giant cells may influence the outcome of [^18^F]FDG-PET studies following radiation therapy. In sum, using a novel radionuclide-based assay capable of single-cell resolution, our study offers novel insights that could refine the interpretation and reliability of [^18^F]FDG-PET scans in patients undergoing radiation therapy.

## Results

### Characterization of giant multinucleated cells following radiation exposure

**Fig. 1:**
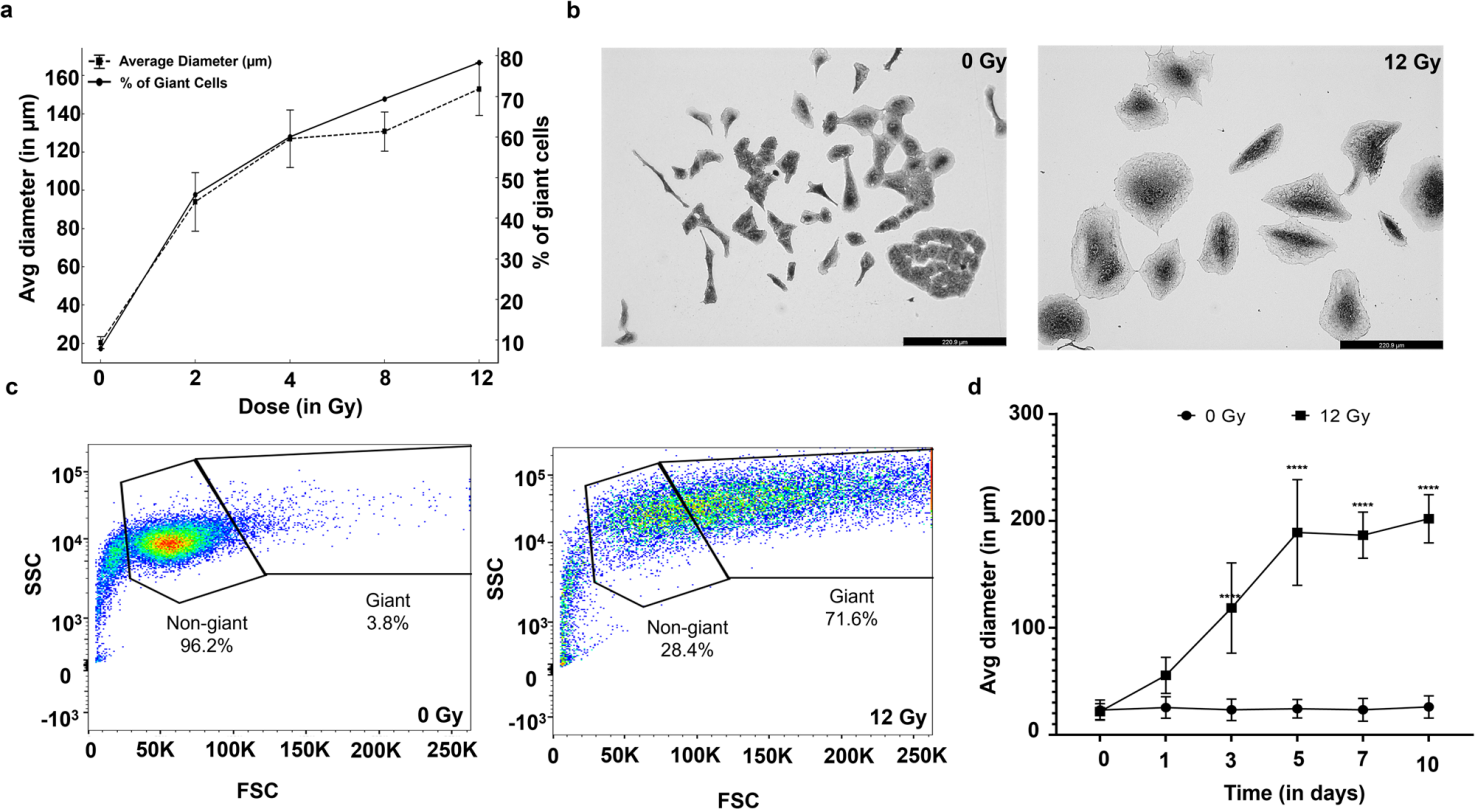
Emergence of the giant cell phenotype following irradiation of A549 cells. **a** Graph showing percentage of giant cells and average diameter of A549 cells, as a function of the radiation dose received. The cells were characterized 3 days after irradiation. **b** Representative brightfield images of unirradiated and irradiated A549 cells, 4 days after 12 Gy irradiation (scale bar, 220 µm). **c** Flow cytometry data showing giant and non-giant populations for both unirradiated and irradiated (12 Gy) A549 cells. **d** Increase in cell size of both unirradiated and 12 Gy irradiated A549 cells over time (****P < 0.0001).

Our first step in this study was to characterize the biological properties of the giant multinucleated cells that arise following radiation exposure. Lung carcinoma cells (A549) were exposed to a range of radiation doses (0–12 Gy), and the size and proportion of the resulting giant cells was measured 72 h later. Cell diameter, measured by optical microscopy, increased in a dose-dependent manner (Fig. 1a). The proportion of giant cells in the irradiated population followed a similar trend. Following this observation, we proceeded with the 12 Gy dosage for subsequent experiments to maximize the yield of giant cell formation.

After receiving 12 Gy, A549 cells became enlarged and multinucleated by day 5, with an average diameter of 210 µm (Fig. 1b). Despite this pronounced transformation, not all A549 cells transitioned into giant cells and a small subset retained their original morphology. To confirm this, we employed flow cytometry to estimate the relative proportion of giant cells on the basis of forward and side scattering. In the absence of radiation treatment, the A549 cultures contained only 3.8% giant cells. In contrast, cells subjected to 12 Gy radiation exhibited a substantial transformation, with 71.6% of the cells being classified as multinucleated giant cells (Fig. 1c). To further elucidate the growth dynamics of these giant cells, we cultured the irradiated cells over 10 days and observed a progressive increase in their size for the initial 5 days, stabilizing thereafter (Fig. 1d).

### Morphological and ploidy analysis of radiation-induced giant cells reveals G2/M cell cycle arrest and senescence

**Fig. 2:**
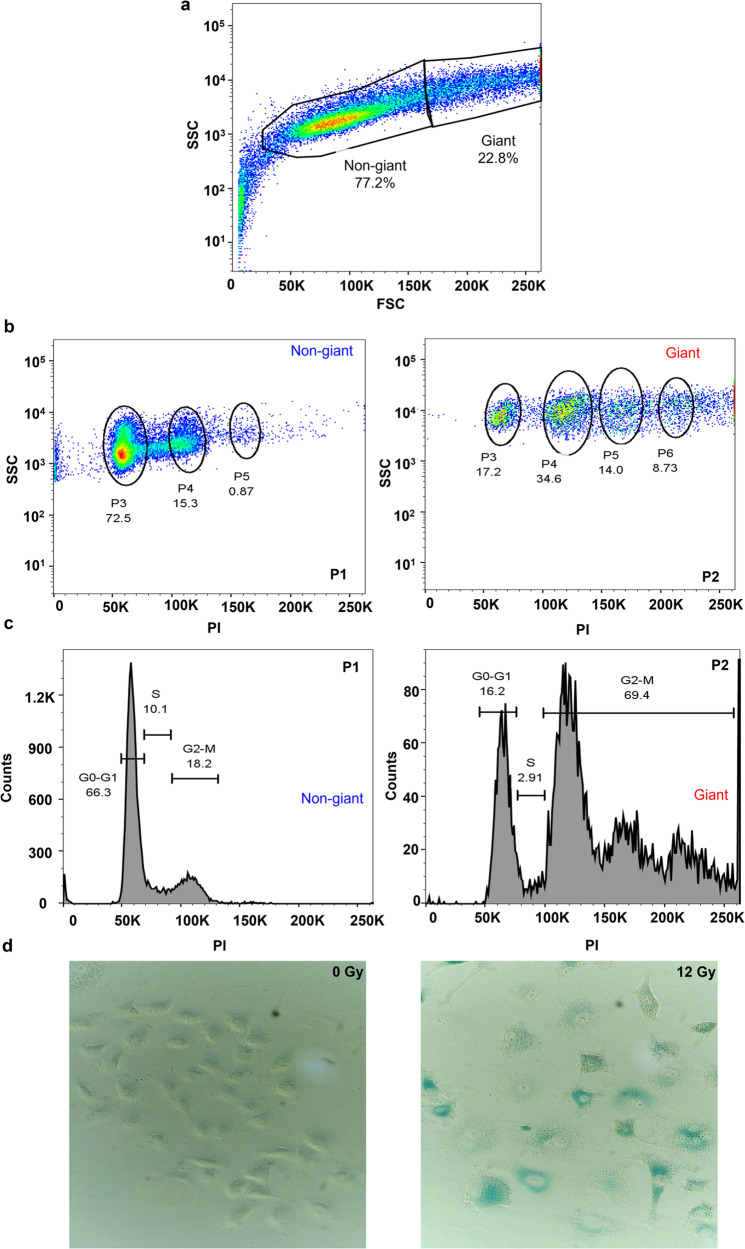
Ploidy and arrest of radiation-induced giant cells. **a** Flow cytometry analysis of 12 Gy irradiated A549 cells showing two different populations i.e. non-giant and giant cells. **b** Ploidy estimation using propidium iodide (PI) staining, for both populations. **c** Cell cycle analysis for both populations. **d** β-gal staining of senescence in both unirradiated and 12 Gy irradiated A549 cells.

Following the morphological assessment of A549 cells post radiation exposure, evidence of multinucleation was distinctly noted. To better understand the ploidy status of these cells, a combination of flow cytometry and propidium iodide (PI) staining was employed. The resultant data (Fig. 2a) highlighted two distinct cell populations 3 days post-irradiation, categorized here as P1 (non-giant, 77.2%) and P2 (giant, 22.8%). Ploidy analysis, based on PI uptake, further divided these populations into smaller subgroups, identified here as P3, P4, P5, and P6 and corresponding to the 2n, 4n, 6n, and 8n states, respectively. Notably, the non-giant cells were primarily in the 2n (72.5%) and 4n (15.3%) state, whereas the giant cell population was more diverse and included cells in the 2n (17.2%), 4n (34.6%), 6n (14.0%), and 8n (8.7%) state.

Analysis of PI staining also provides insight into the distribution of cells along the different phases of the cell cycle. In the non-giant population of irradiated A549 cells, 66.3% of cells were localized in the G0-G1 phase and 18.2% in the G2-M phase. In the giant population, 16.2% of cells were in the G0-G1 phase, and a pronounced 69.4% in the G2-M phase, signaling a predominant G2-M cell cycle arrest (Fig. 2c). These findings led us to investigate cellular senescence as a potential contributor to the observed phenotype. Using β-galactosidase staining, we found that 84.6% of the irradiated cells exhibited signs of senescence, compared to 8.4% for control unirradiated cells (*P* < 0.0001; Fig. 2d). Collectively, these observations indicate that, after receiving 12 Gy, A549 cells exhibit polyploidy and are anchored in the G2/M phase, with radiation-induced senescence being a plausible mechanism underlying this arrest.

### Metabolic profiling of radiation-induced giant cells demonstrates elevated [^18^F]FDG uptake

Having demonstrated the unique morphological and chromosomal characteristics of radiation-induced giant cells, we shifted our focus to their metabolic characteristics. Given their enlarged size, we postulated that these giant cells would have increased demand for glucose. To test this hypothesis, we developed a novel single-cell analysis workflow to characterize the metabolic flux of [^18^F]FDG of both irradiated (12 Gy) and unirradiated A549 cells. Approximately 1 × 10^5^ A549 cells were incubated for 1 h with [^18^F]FDG at a concentration of 185 MBq/ml in a total volume of 2 ml, then dispensed as single cells into individual microcentrifuge tubes using a microfluidics cell dispenser (Namocell, USA). The radioactivity of individual cells was then measured using an automated gamma counter.

**Fig. 3:**
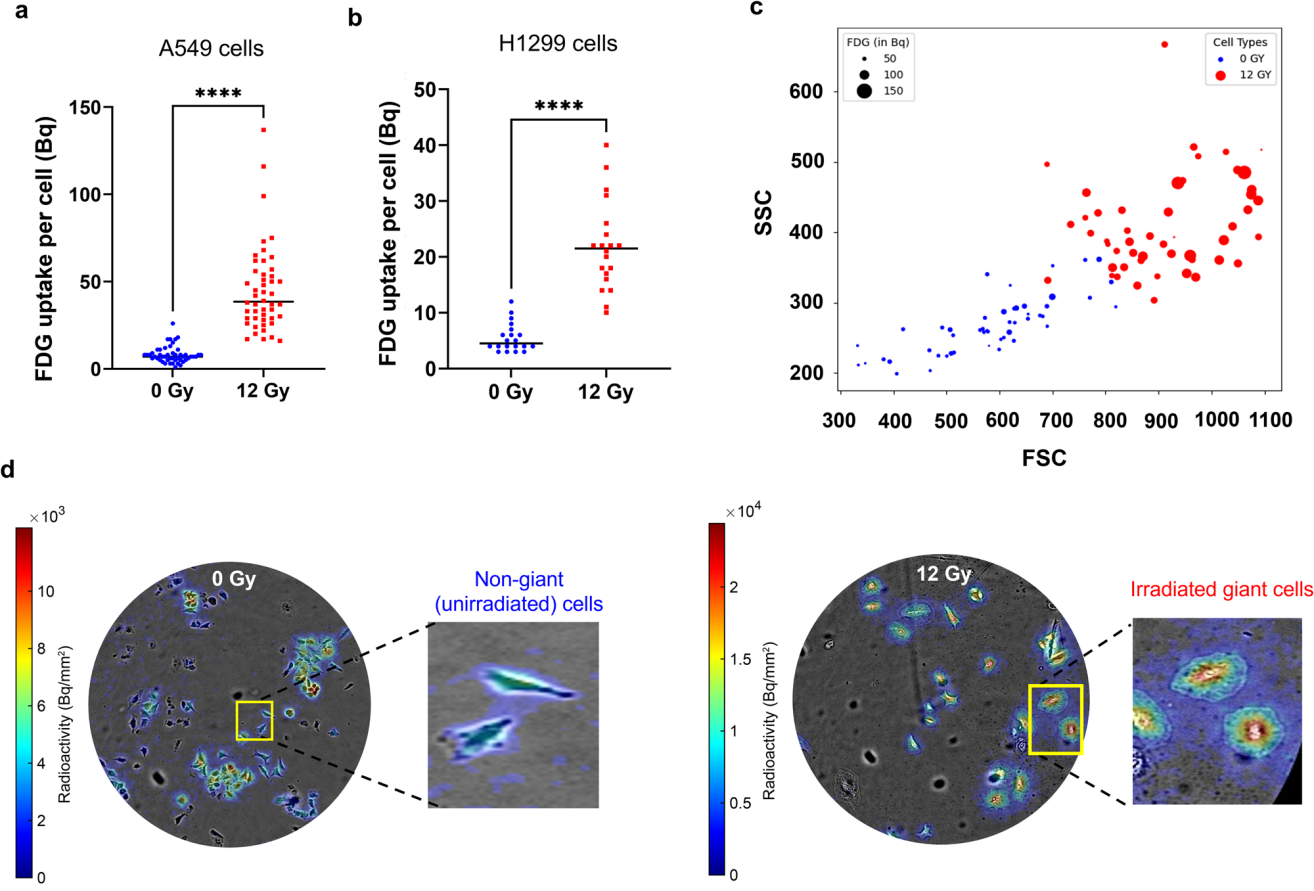
Radiation induced giant cells have increased [^18^F]FDG uptake. **a** Single-cell [^18^F]FDG uptake measured by gamma counting after single-cell dispensing. Data was acquired 5 days after irradiation (12 Gy) of A549 cells (****p < 0.0001). **b** Same experiment, was performed using H1299 cells irradiated with 6 Gy (****p < 0.0001). **c** The data in **a**, plotted to highlight the correlation between forward scattering (FSC) representing size, side scattering (SSC) representing granularity, and [^18^F]FDG uptake. The diameter of the solid bubbles represents [^18^F]FDG uptake in Bq of single cells. **d** Radioluminescence microscopy imaging of non-giant (unirradiated) and giant cells (12 Gy irradiated). Sections of the image are magnified to depict the radioluminescence in individual cells.

Using this workflow, we observed that [^18^F]FDG uptake increased approximately 4-fold relative to control cells (*P* < 0.0001). On average, irradiated cells took up 45 Bq/cell, compared to 8 Bq/cell for unirradiated cells (Fig. 3a). The uptake was heterogeneous in both groups, as reflected by a coefficient of variation of 54% and 58% for the irradiated and control cells, respectively. To corroborate these observations, we repeated this experiment using the H1299 cell line (human non-small cell lung carcinoma). In contrast to A549 cells, H1299 cells exhibit greater radiosensitivity and formed giant cells at a radiation dose of 6 Gy. As shown in Fig. 3b, single H1299 cells had significantly increased [^18^F]FDG uptake when exposed to radiation (*P* < 0.0001), although overall uptake levels were lower than those observed in A549 cells. This evidence underscores that the enhanced [^18^F]FDG uptake by radiation-induced giant cells is consistent across at least two different lung cancer cell types.

In addition, we investigated single-cell [^18^F]FDG uptake in relation to forward scatter (indicative of size) and side scatter (reflecting granularity). The results, shown in Fig. 3c, reveal a notable association between uptake and forward scatter, indicative of a link between cell size and [^18^F]FDG metabolic activity. Upon analyzing the correlation between [^18^F]FDG, FSC and SCC, our analysis revealed that in giant cells, increased [^18^F]FDG uptake has a significant positive correlation with cell size (FSC), as evidenced by a Pearson correlation coefficient of 0.38 (*P* = 0.007). However, there was no significant correlation observed with respect to granularity (SSC). In control cells, [^18^F]FDG uptake was weakly correlated with cell size (Pearson correlation coefficient of 0.25; *P* = 0.08). These results suggest that, in giant cells, higher [^18^F]FDG uptake may be driven by the larger cell volume.

To further substantiate these findings, we leveraged radioluminescence microscopy (RLM), a method capable of visualizing single-cell [^18^F]FDG uptake in vitro^9,10^. Figure 3d portrays the pronounced [^18^F]FDG uptake of radiation-induced giant cells relative to unirradiated control cells and corroborates our single-cell gamma-counting measurements. Collectively, our data underscores the metabolic vitality of these radiation-induced giant cells. Despite their arrested cellular proliferation, they manifest heightened metabolic activity, evidenced by their elevated [^18^F]FDG uptake relative to unirradiated A549 cells.

### Radiation-induced metabolically active multinucleated giant cell in xenograft mouse model

**Fig. 4:**
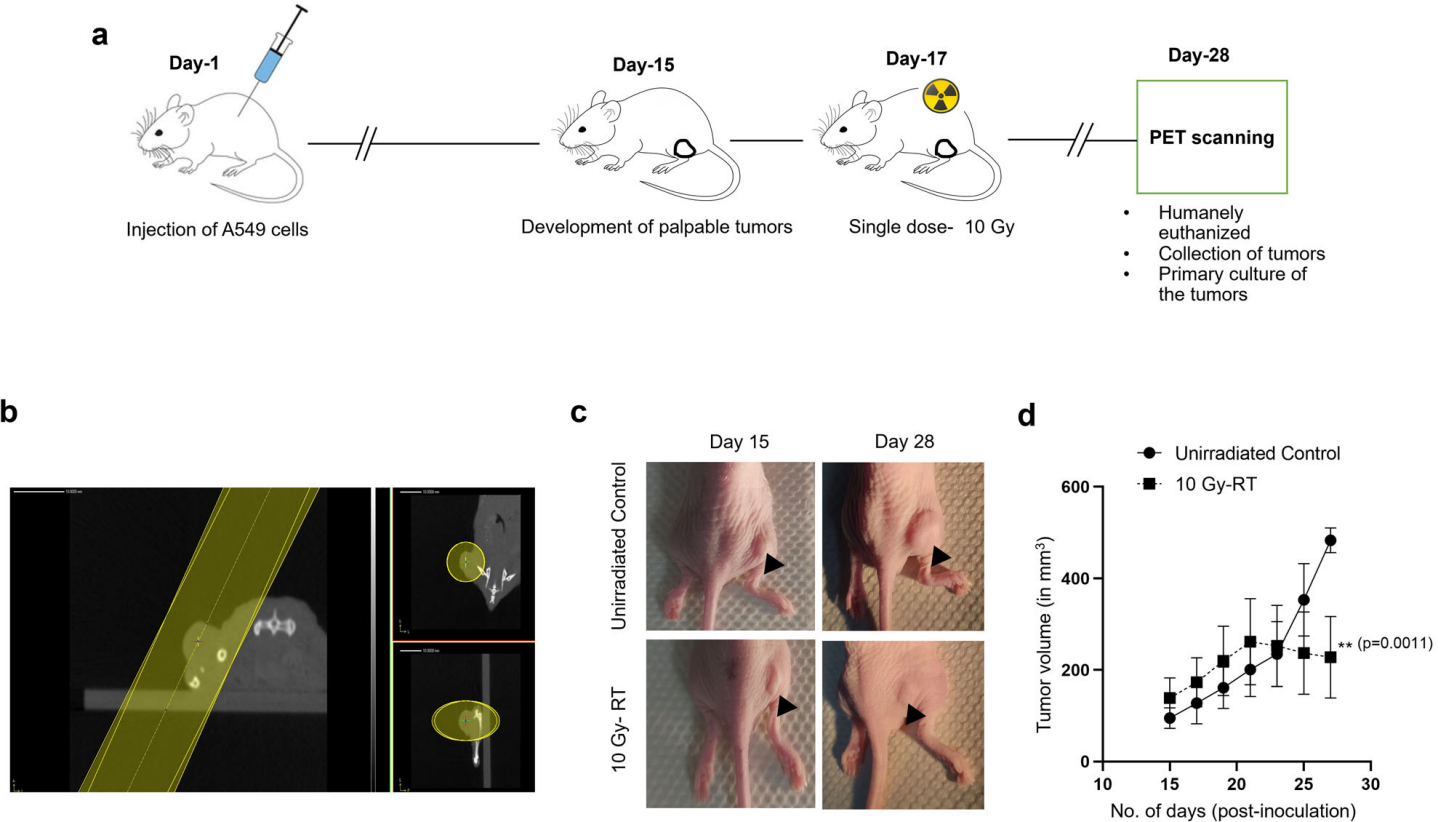
In vivo experimental plan and tumor generation in nude mice. **a** Schematic representation of the experimental timeline. **b** Representative radiation treatment plan for tumor bearing mice, showing two opposing beams in yellow. **c** Representative photographs of tumor bearing mice from the treated (*n* = 3) and untreated (*n* = 2) groups, taken 15 and 28 days post irradiation. The black arrows show the tumor location. **d** Change in tumor volume over time for both groups.

To evaluate the applicability of our findings to a more clinically relevant setting, we extended our investigation to an in vivo xenograft mouse model (Fig. 4a). Palpable tumors were observed in mice 15 days after the subcutaneous inoculation of A549 cancer cells. On day 17, these tumor-bearing mice received a single 10 Gy dose of conformal radiation to the tumor, with a separate unirradiated cohort serving as controls. The effect of the radiation treatment on the metabolic activity of the tumor was assessed 11 days after irradiation using in vivo PET imaging followed by ex vivo single-cell gamma counting.

**Fig. 5:**
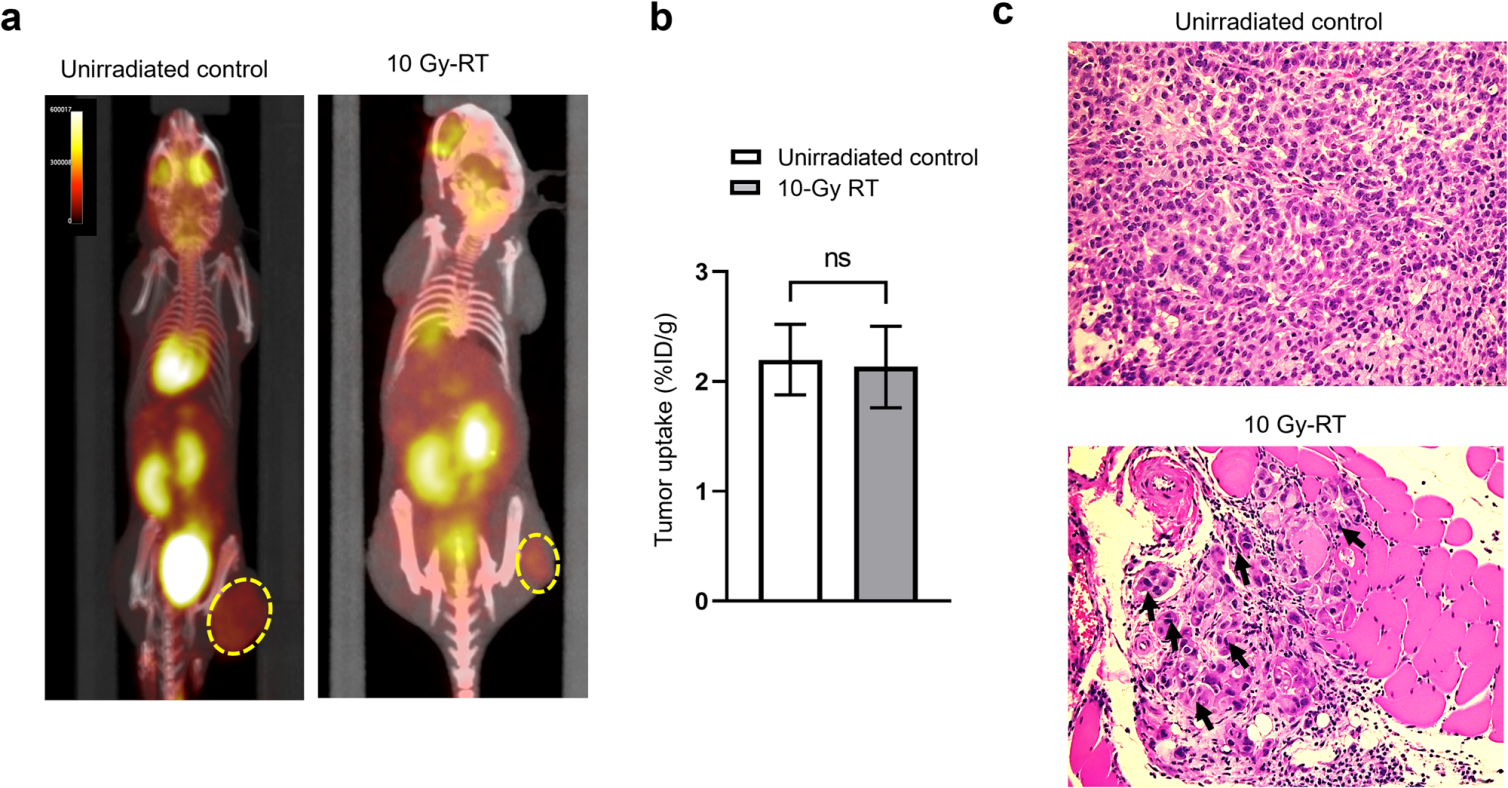
Emergence and [^18^F]FDG uptake of giant cells in vivo. **a** Representative PET-CT imaging of both unirradiated and 10-Gy treated mice. **b** [^18^F]FDG tumor uptake (shown as a percentage of the injected dose per tumor mass) in both groups (ns, not significant). **c** Representative H&E staining of the extracted tumors for both groups. The black arrowheads denote the presence of multinucleated giant cells in the tissue samples.

As could be expected, radiation therapy halted tumor progression, leading to 56% smaller tumors in the treated group relative to the control group (*P* = 0.0011; Fig. 4b–d). However, [^18^F]FDG uptake was not significantly affected, with values for the control group at 2.3 ± 0.7% ID/g and those for the 10 Gy cohort at 2.1 ± 1.1% ID/g (Fig. 5a, b). Thus, despite being smaller, the irradiated tumors still maintained similar level of [^18^F]FDG uptake as their untreated counterparts.

**Fig. 6:**
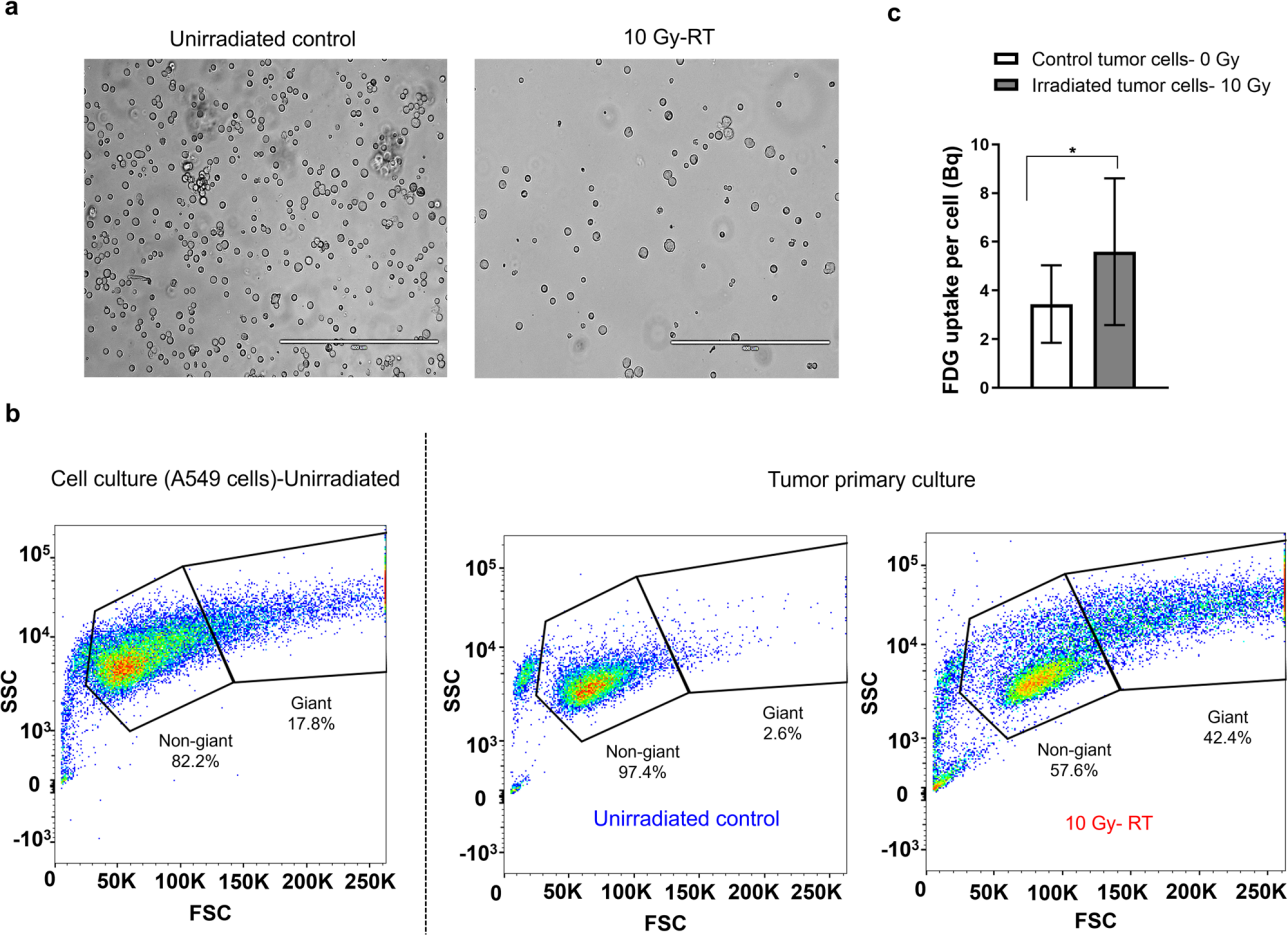
Primary cells cultured from the irradiated tumors show higher [^18^F]FDG uptake. **a** Representative brightfield images of primary cells cultured from excised tumor mass for both unirradiated and irradiated animals. **b** Flow cytometry analysis of cells from the A549 cell lines and from primary culture of cells extracted from untreated and treated tumors. The gating parameters were defined using the in vitro cell line as a reference. **c** [^18^F]FDG uptake of primary single cells extracted from unirradiated and irradiated tumors (*p < 0.05).

Following this analysis, the tumors were excised for further histological examination and primary culture. Hematoxylin and eosin staining highlighted the emergence of multinucleated giant cells (black arrows), but only in the irradiated samples (Fig. 5c). After harvesting the primary tumor cells, morphological assessments under a brightfield microscope revealed a heterogeneous mix of giant and non-giant cells in the 10 Gy cohort (Fig. 6a). Using flow cytometry analysis and gating parameters derived from unirradiated A549 cells cultured in vivo, we further quantified this heterogeneity and found that the proportion of giant cells was far higher in irradiated tumors (42.4%) than unirradiated tumors (2.6%; Fig. 6b). This result follows the same trend as previously observed in vitro, although the proportion of giant cells was higher in the former case.

Moving our focus to the metabolic properties of these cells, we then used gamma counting to measure the [^18^F]FDG uptake of individual cells obtained by dissociating A549 tumors into single-cell suspensions. Primary cells from irradiated A549 tumors took up significantly more [^18^F]FDG (average uptake of 6.2 Bq) than cells from unirradiated tumor (average uptake of 3.4 Bq; *P* = 0.011; Fig. 6c), reflecting an increase in metabolic activity that was however less pronounced than previously observed with in vitro cells. Taken together, in vivo observations corroborate in vitro findings: radiation induces multinucleated giant cells within tumors, which have increased avidity for [^18^F]FDG.

## Discussion

The deployment of [^18^F]FDG-PET imaging in oncology has helped decipher the complex metabolic patterns of tumors, yet because of limited resolution, significant gaps persist in our understanding of these processes at the cellular level, particularly in the context of radiation-induced changes. While [^18^F]FDG-PET imaging provides a proxy for bulk tumor metabolism, the heterogeneity of cellular responses to ionizing radiation poses a challenge in interpreting these images accurately^12–14^. This is especially true in the context of tumors, where the metabolic behavior of cancer cells can significantly deviate from the norm. Our study addresses this crucial gap by exploring the [^18^F]FDG uptake of radiation-induced multinucleated giant cells. Understanding these dynamics is pivotal for refining our interpretation of medical images in oncology.

This study represents a significant advancement in understanding the metabolic behavior of cancer cells in response to ionizing radiation, focusing on the transformation of A549 human lung cancer cells into multinucleated giant cells and the effects of this transformation on [^18^F]FDG uptake. This research, by employing a novel methodology combining a microfluidics-based cell-sorter and an automated gamma counter, provides some vital insights into the [^18^F]FDG uptake of single cancer cells after radiation. The transformation of A549 cells into multinucleated giant cells following a 12 Gy dose of ionizing radiation aligns with previous literature^5,6,15^. However, our approach offers a deeper understanding of this phenomenon. This transformation is not merely a cellular anomaly but might reflect an adaptive mechanism to DNA damage by radiation. While previous studies^6,15^ have noted the emergence of such cells post-irradiation, our research stands out in its ability to measure their metabolic activity at a more granular level. The correlation between elevated ploidy, increased cell size, and augmented metabolism adds a new dimension to our understanding of post-radiation cellular behavior.

Following in vivo tumor irradiation, A549 cells became larger and took up significantly more [^18^F]FDG than unirradiated tumor cells. In contrast, irradiated tumors were smaller and had levels of uptake similar to unirradiated tumors. This apparent contradiction is best understood considering that, post radiation, the tumor mass comprises both live and dead cells. The uptake of FDG by the tumor is a balance between the lack of uptake of dead cells and the increased uptake of surviving giant cells. Therefore, an essential implication of our work is that FDG uptake by tumors does not necessarily mirror the count of viable cells, since the killing of tumor cells can be counterbalanced by the increased uptake of the surviving cells.

Several research efforts have explored the increased glucose requirements and [^18^F]FDG uptake in tumor cells following radiation therapy. One such report noted a temporary surge in the glycolytic pathway in LS180 human colon adenocarcinoma cells after radiation^16^. Similarly, another study observed an increase in [^18^F]FDG uptake in irradiated human tumor xenografts^17^. Earlier investigations, such as those by Schmidtke in 1998^7^ and Higashi in 1993^8^, also found heightened [^18^F]FDG uptake post-radiation, though their methods primarily focused on aggregate data, thus missing the variability among individual cells. Our study, however, employs a different methodology, assessing each cell individually. This approach unveils a wide spectrum of interdependent metabolic behaviors and responses to radiation in these cells. The use of a microfluidics-based cell-sorter combined with an automated gamma counter allowed us to analyze both [^18^F]FDG uptake and cell size on a single-cell level, marking a significant departure from traditional bulk cell analyses, which lump all the cells into a single average population. These traditional methods often overlook the intricate metabolic variations at the single-cell level, leading to a generalized and sometimes inaccurate understanding of cellular responses to treatments like radiation therapy. Our novel approach, which combines the precision of microfluidics-based cell-sorting with the sensitivity of an automated gamma counter, overcomes these limitations. By allowing for the detailed analysis of [^18^F]FDG uptake in individual cells, we can uncover metabolic patterns that would otherwise remain hidden in bulk cell analyses. This level of detail is critical in understanding the heterogeneity of cancer cell responses and refining therapeutic strategies accordingly.

Cancer cells have been shown to have enhanced glucose needs following exposure to radiation. In lung cancer, for instance, A549 cells surviving post-irradiation display a marked increase in glucose uptake and upregulation of the GLUT1 glucose transporter^18^. Although the exact mechanism is not yet fully understood, increased cytoplasmic volume and polyploidy may contribute to this transformation. Beyond radiation therapy, multinucleated giant cells implicated in diseases like schistosomiasis, sarcoidosis, and arthritis require substantial energy for their formation, especially for membrane fusion and cytoplasmic expansion, thereby necessitating increased glucose uptake^19^. Additionally, chemotherapy-induced polyploid giant cancer cells undergo significant metabolic shifts post-treatment, indicating altered metabolic pathways, including those related to glucose metabolism^20^. These insights highlight the broader metabolic requirements of giant cells, underscoring their adaptive responses to diverse stressors.

The relationship between ploidy status and glucose uptake is another aspect of cellular metabolism warranting attention. Increased ploidy often results in enlarged cellular and nuclear volumes, leading to altered metabolic states and genomic instability^21^. Studies on budding yeast (*Saccharomyces cerevisiae*) have revealed a linear increase in cell volume with ploidy whereas protein biosynthesis scales sublinearly^22^. While there is a general hypothesis that polyploid cells might have higher metabolic demands, including an increased need for glucose, the specific relationship between polyploidy and glucose requirements is not straightforward and depends on various factors such as cell type, the extent of polyploidy, and the cellular environment. A study focusing on human aortic endothelial cells demonstrated that polyploid cells exhibit the characteristics of endothelial dysfunction, and their accumulation was accelerated during growth in high glucose media, suggesting a complex interaction between polyploidy and glucose utilization^23^. Therefore, in the context of our study, it can be speculated that polyploidy might contribute to increasing metabolic needs and which in turn may lead to higher glucose uptake per cell.

An additional observation made in this study was the predominance of G2/M phase arrest in the giant cells that arose following irradiation of A549 lung cancer cells. This aligns with existing literature indicating that radiation can induce G2/M phase arrest as a cellular response mechanism^24–26^. Further expanding this understanding, a previous study using radioluminescence microscopy yielded new insight into the relationship between cell cycle phase and [^18^F]FDG uptake. In their research on HeLa cells, Sung et al. demonstrated that cells in the S, G2, or M phases take up twice as much [^18^F]FDG than those in the G1 phase^27^. Although focused on HeLa cells, their study offers a pertinent perspective that aligns with our observations in radiation induced A549 giant cells. The increased [^18^F]FDG uptake associated with the S, G2, or M phases, as observed by Sung et al., mirrors the metabolic patterns we noted in the G2/M arrested giant cells.

Collectively, our findings, in conjunction with these previous reports, reinforce the metabolic implications of cell cycle alterations post-radiation. This parallel not only validates our observations but also deepens our understanding of the metabolic behavior of single cancer cells under radiation stress, offering a more comprehensive view of the intricate interplay between cellular metabolism, the cell cycle, and radiation response.

The enhanced FDG uptake in giant cells post-radiation, as revealed in our study, has significant implications for FDG-PET imaging in clinical oncology. Traditionally, FDG-PET imaging is used to assess metabolic activity in tumors, with the assumption that higher [^18^F]FDG uptake correlates with aggressive tumor progression. Our findings challenge this assumption by showing that radiation-induced giant cells, which are not actively proliferating, can also exhibit high [^18^F]FDG uptake. This insight is crucial for accurate reading of post-treatment [^18^F]FDG-PET studies, where the metabolic signals of these non-dividing yet metabolically active cells could lead to potential misinterpretations.

## Conclusion

Using a novel analytical workflow for assessing single-cell [^18^F]FDG uptake, our research provides compelling evidence that ionizing radiation has a significant impact on human lung cancer cells, notably by inducing the formation of metabolically active multinucleated giant cells. Despite the apparent cessation of cellular division, the metabolic vigor of these cells remains undiminished. This phenomenon is intriguing, as one might assume that cells rendered incapable of further division might exhibit decreased metabolic activity. However, our findings indicate the contrary: these giant cells continue to actively metabolize glucose, a process evidenced by their contribution to the measured [^18^F]FDG-PET signal. This discovery raises critical considerations for oncological imaging. [^18^F]FDG-PET, a mainstay in cancer diagnostics and treatment monitoring, might project misleading information due to the persistent metabolic activity of these non-proliferating cells. As such, when interpreting [^18^F]FDG-PET images, clinicians should be cautious, recognizing the potentially confounding influence of these radiation-induced giant cells. Ultimately, this study underscores the necessity to refine our understanding and interpretation of post-therapy imaging, ensuring that treatment strategies are both informed and effective.

## Materials and methods

### Cell culture

A549 lung adenocarcinoma cells, procured from the American Type Culture Collection (ATCC), were cultured using standard Dulbecco’s Modified Eagle Medium (DMEM) (ThermoFisher Scientific, #11995065) supplemented with 10% fetal bovine serum (ThermoFisher Scientific, #26140079), 4 mM L-glutamine, 1 mM sodium pyruvate, 100 IU of penicillin, and 100 μg/mL of streptomycin. Initially the cells were cultured in standard T-25 tissue culture flasks within a humidified incubator set at 37 °C with 5% CO_2_. Upon reaching 70% confluence, they were trypsinized and used in appropriate cell culture plates for further experimental conditions. H1299 lung cancer cells were a kind gift from Dr. Maximilian Diehn (Professor, Radiation Oncology, Stanford University, USA). These cells were maintained in conditions similar to those used for A549 cells, with the exception that the culture medium utilized was RPMI-1640 Medium (Sigma-Aldrich, #R8758).

### Cell irradiation

A549 and H1299 cells were cultured and then irradiated in T-25 flasks. Different doses (2, 4, 6, 8, 10, and 12 Gy) were imparted to the cultured cells using a cabinet X-ray irradiator (X-Rad SmART; Precision X-ray Inc.) operating at 225 kVp and 13 mA, using the standard 0.3 mm Cu filter and no collimator. Dosimetry for the set-up was confirmed using an ion chamber according to the standard TG61 protocol.

### Radioluminescence microscopy (RLM) imaging

Radioluminescence microscopy (RLM) was used as previously described^28,29^. In brief, before imaging, A549 cells were seeded on CdWO_4_ scintillators (1 cm × 1 cm × 0.5 mm, with both sides polished, sourced from MTI Inc.). Twenty-four hours after seeding, the cells were incubated in glucose-free DMEM medium enriched with 10% FBS for 1 hr at 37 °C in a 5% CO_2_ incubator. Thereafter, [^18^F]FDG (20 MBq/mL) was added to the dish and cellular uptake of the tracer was allowed to proceed for another 1 hr under the same conditions. To eliminate any residual [^18^F]FDG, the cells were then washed thrice using cell-culture grade PBS. Following these preparatory steps, the cellular uptake of [^18^F]FDG was imaged using RLM. Sequential images of individual ionization tracks were captured with an EM-CCD (Hamamatsu Image EMC9100-14) set at maximum gain, an integration time ranging of 20 ms, 10,000 frames, and 2 × 2 binning. The chosen integration time averaged around 40–50 decay events per frame. Reconstructed RLM images were generated using our specific methodology, termed optical reconstruction of beta-ionization tracks, as discussed earlier^30^.

### Cell labeling using [^18^F]FDG

Clinical-grade [^18^F]FDG was synthesized at the Stanford Cyclotron and Radiochemistry Facility. To ensure higher activity, the tracer was used within 3–4 h of production. A549 cells were initially seeded in a 6-well plate at a density of 1 × 10^5^cells/well, then incubated for 48 h at 37 °C and 5% CO_2_. Before treating the cells with [^18^F]FDG, cells were placed for 1 h in glucose-free medium (DMEM supplemented with L-Glutamine; Thermo Fisher Scientific, #11966025) containing 1% FBS. Thereafter, cells were switched to fresh glucose-free media supplemented with [^18^F]FDG at a concentration of 20 MBq/ml. After a 1-hour [^18^F]FDG incubation, cells were rinsed thrice with 2 ml of PBS to eliminate any residual [^18^F]FDG.

### Single-cell dispensing

Microfluidic-based single-cell sorting was executed using the Hana single-cell sorter from Namocell, USA. Preceding this, cells underwent staining with Calcein-AM (2.5 nM; ThermoFisher Scientific, #65085381) for 15 min. Per the instrument’s protocol, a concentration of 5000 cells/ml was employed for each condition, and about 500 µl of the cell suspension was loaded into the microfluidic cartridge. Dual gating was applied for cell collection. Primarily, FSC (forward scattering) and SSC (side scattering) were used to gate unirradiated A549 cells, filtering out debris and retaining intact cells. The secondary gate selected FITC-positive cells, ensuring the collection of live cells. For dispensing of radiation-induced giant cells, both FSC/SSC and FITC gating parameters were adjusted to account for their larger size. Each cell was dispensed in a droplet measuring 1 μl into a 96-well tissue culture plate, which was prefilled with 100 µl DMEM media per well.

### Single-cell gamma counting

The Hidex gamma counter system was employed to quantify the radioactivity of single cells. Radiolabeled single cells were organized into racks for the automated counting procedure. Every tube was supplemented with single cells suspended in 100 μl of fresh media. The tubes with only media were used as a blank to calculate the background radioactivity. The radioactivity of individual cells was measured using a counting time of 1 min and expressed in counts per second, later translated to Bq based on cross-calibration to a medical dose calibrator.

### Tumor xenograft model

All animal experiments received approval from Stanford University’s Administrative Panel on Laboratory Animal Care under protocol #28882. Five female Nu/Nu mice were sourced from Charles River Laboratories. Two mice were used as control (without irradiation) and 3 mice were used as treated group (10 Gy irradiation). A549 cells were freshly collected and resuspended in a 1:1 mix of matrigel and PBS, then 1 × 10^6^ cells (50 µL) were subcutaneously injected into the thigh region. Tumors were then allowed to develop over a period of 2 weeks, then were treated with radiation therapy.

### Radiation therapy of tumor xenografts

Radiation therapy was delivered as a single fraction of 10 Gy using an image-guided conformal irradiator (SmART, Precision X-ray Inc). Each treated mouse was imaged using cone-beam CT prior to treatment to ensure the correct positioning of the tumor within the radiation beam. The radiation treatment was delivered using 2 opposing beams (225 kVp, 13 mA, and 0.3 mm Cu filtration) collimated with 10 cm circular apertures and was planned using RT_Image software. Mice were anesthetized (2% isoflurane) during these procedures.

### Micro PET/CT scanning

[^18^F]FDG-PET imaging was conducted 10 days after irradiation. Food was withheld from mice for 12 h prior to imaging. Animals were anesthetized with 3% isoflurane gas and then received an intravenous injection of 20 MBq [^18^F]FDG. Afterwards, animals remained under anesthesia for 1 h to avoid muscle uptake, then were imaged using a GNEXT PET/CT scanner (Sofie Biosciences). We captured PET images over a span of 10 min, utilizing the conventional energy window of 350–650 keV. CT scans were taken using default parameters, with a scan duration of 2 min and a beam energy of 80 kVp. The images were reconstructed using standard OSEM and analyzed using the Amide software.

### Primary cell culture

Following PET imaging, the mice were euthanized, and tumors were collected and washed with PBS before being surgically divided into two halves. One half was stored in 10% neutral buffered formalin for H&E staining and the other half was used for primary culture. The tumor tissue sample was finely chopped using a scalpel and placed into ice-cold Trypsin. The mixture of tumors and trypsin was then warmed to 37 °C and vortexed for 30 minutes. The resulting solution was filtered through a 40-micrometer strainer to isolate individual cells, followed by centrifugation. The collected cells were then resuspended in growth media and seeded onto T-25 cell culture flasks.

### Propidium Iodide (PI) staining

Both 12 Gy irradiated and unirradiated A549 cells were seeded onto T-25 cell culture flasks. Five days later, the cells were trypsinized and treated with 50 μL of RNAse solution (stock concentration of 100 μgmL − 1). After 30 min of incubation, they were fixed with 70% chilled ethanol for 1 h at 4 °C and then stained with PI at a concentration of 50 µg/ mL. Cell cycle phase was estimated after a reading was acquired by flow cytometry (BD FACSverse™, CA, USA) and appropriate gating was carried out.

### β-gal staining

A549 cells, both irradiated with 12 Gy and unirradiated, were seeded onto 35 mm culture dishes and allowed to grow for 5 days. The cells were then washed twice with 1× PBS and fixed with 4% paraformaldehyde for 10 min at room temperature. Following fixation, the cells were treated with a β-gal staining solution, which was prepared using 0.1% X-gal, 5 mM of both potassium ferrocyanide and potassium ferricyanide, 150 mM sodium chloride, and 2 mM magnesium chloride, all dissolved in a 40 mM citric acid/sodium phosphate buffer (pH 6.0). The cells were then incubated overnight at 37 °C without CO_2_. The following day, the cells were rinsed and resuspended in 1× PBS. An inverted light microscope (Leica, DMi8) was used to image the cells.

### Statistics

Statistical differences between both groups were calculated using an unpaired, 2-tailed Student t-test. The level of significance is indicated as follows: *P < 0.05,**P < 0.01,***P < 0.001,****P < 0.0001. Statistical analyses were performed using the Prism software (version 9.0; GraphPad, SanDiego, CA).

## Data Availability

No datasets were generated or analysed during the current study.
